# Loss of DNA-PK complex in recurrent pterygium suggests a role for defective double-strand break repair

**DOI:** 10.1007/s00417-026-07283-4

**Published:** 2026-05-26

**Authors:** Leonardo Lupacchini, Marco Marenco, Marco Rosina, Fabiana Mallone, Cristiana Mollinari, Angelina Pernazza, Alessandro Lambiase, Daniela Merlo

**Affiliations:** 1 Laboratory of Molecular and Cellular Neurobiology, IRCCS San Raffaele Roma, Via di Val Cannuta 247, Rome, 00166 Italy; 2 Department of Sense Organs, Sapienza - University of Rome, Viale del Policlinico 155, Rome, 00161 Italy; 3 Department of Neuroscience, Italian National Institute of Health (ISS), Viale Regina Elena 299, Rome, 00161 Italy; 4 IRCCS-G.B. Bietti Foundation, Via Livenza 3, Rome, 00198 Italy; 5 Institute of Translational Pharmacology (IFT), National Research Council, Via Fosso del Cavaliere 100, Rome, 00133 Italy; 6 Department of Medico-Surgical Sciences and Biotechnologies, Sapienza University of Rome, Corso della Repubblica 79, Latina, 04100 Italy

## Abstract

**Purpose:**

Pterygium is a common ocular surface disorder strongly associated with ultraviolet (UV) radiation exposure and oxidative stress. Double-strand breaks (DSBs) are among the most deleterious forms of DNA damage, and their repair mainly relies on the non-homologous end joining (NHEJ) pathway, in which the DNA-dependent protein kinase (DNA-PK) complex plays a central role. This study investigated the expression of the DNA-PK complex components—DNA-PKcs, Ku70, and Ku86—in pterygium compared with healthy conjunctiva.

**Methods:**

Conjunctival tissues were obtained from six patients undergoing surgical excision of recurrent pterygium and 6 age- and sex-matched controls undergoing ocular surgery without surface disease. Protein and transcript expression of DNA-PKcs, Ku70, and Ku86 were assessed by immunofluorescence, Western blotting, and real-time PCR.

**Results:**

Immunofluorescence revealed markedly reduced DNA-PKcs immunoreactivity in the epithelium of pterygium conjunctiva compared with marked staining in controls. Western blot analysis demonstrated a significant decrease of DNA-PKcs protein, paralleled by a ~ 13-fold reduction of its mRNA. Similarly, Ku70 and Ku86 protein levels were dramatically reduced (by ~ 93.5% and ~ 92.0%, respectively) in pterygium compared with controls.

**Conclusions:**

This study provides the first evidence of profound down-regulation of the DNA-PK complex in pterygium. The loss of DNA-PKcs and its regulatory subunits Ku70 and Ku86 suggests impaired DSB repair capacity, potentially driven by chronic UV-induced oxidative stress. These alterations may contribute to pterygium pathogenesis and recurrence, highlighting DNA-PK complex proteins as potential biomarkers of disease susceptibility and progression.

**Supplementary Information:**

The online version contains supplementary material available at https://doi.org/10.1007/s00417-026-07283-4.

## Key Message

What is known

• Pterygium is a frequent ocular surface disorder strongly associated with ultraviolet radiation exposure and oxidative stress.

• DNA-PK activity is highly affected by oxidative stress and other genes involved in Double Strand Break Repair are differentially expressed in pterygium.

What is new

• This study provides the first evidence that impaired Double Strand Break Repair mechanism is associated with pterygium.

• The DNA-PK complex protein expression may be potential biomarker of pterygium susceptibility and progression.

**Supplementary Information:**

The online version contains supplementary material available at https://doi.org/10.1007/s00417-026-07283-4.

## Introduction

Pterygium is a common ocular surface disorder characterized by fibrovascular, wing-shaped overgrowth of the conjunctival tissue that extends to the cornea and may lead to visual impairment in advanced stages [1]. 

It is a relatively common ocular surface condition, with epidemiological studies reporting prevalence rates ranging from 2.83% in Australians to as high as 39.0% among the Chinese Bai population [2, 3]. Overall, pterygium is estimated to affect approximately 22% of populations in countries located near the equator, while its prevalence drops below 2% in regions farther from the equator [2]. These marked epidemiological differences strongly indicate that environmental factors play a critical role in the development of the disease [2, 4]. 

Specifically, the consistent association between pterygium and prolonged sunlight exposure supports the hypothesis that ultraviolet (UV) radiation is a major environmental factor in its pathogenesis. As a result, pterygium is sometimes referred to as ‘ophthalmoheliosis’—a sun-induced ocular condition [2, 4–7]. 

Research indicates that UV light exposure may influence the development of pterygium either directly by phototoxic effect, or indirectly by formation of radical oxygen species (ROS) that cause oxidative DNA damage. Moreover, UV radiation has been demonstrated to alter the methylation in DNA sequences related to matrix remodeling and cell adhesion [4, 8]. These processes can produce a wide range of DNA damage, including replication-blocking adducts and strand breaks [4, 6, 9–11]. 

Among these, DNA double-strand breaks (DSBs) are the most severe form of genomic damage that leads to mutations and genome instability [12–14]. 

There are two mechanisms for DSB repair (DSBR), consisting of non-homologous end joining (NHEJ) and homologous recombination (HR) [15–17]. The choice between these mechanisms is largely dictated by the cell cycle phase and the availability of specific repair proteins [15–17]. Although traditionally viewed as distinct, accumulating evidence indicates that NHEJ and HR can be concurrently active and may even cooperate under certain conditions [15–18]. NHEJ is an error-prone mechanism that ligates DNA ends with minimal or no sequence homology, and remains active throughout all phases of the cell cycle. In contrast, HR is an error-free repair process that utilizes a sister chromatid as a template for repair, thereby restricting its activity to the S and G2 phases of the cell cycle [15–17]. 

In humans, NHEJ is the predominant pathway of DSBR and involves five proteins: namely, the ligase IV (LIG4), its associated protein XRCC4, and the three components of the DNA dependent protein kinase (DNA-PK) complex, Ku70, Ku80/Ku86, and the catalytic subunit DNA-PKcs. Specifically, Ku70 and Ku80 constitute the Ku heterodimer and consist of two subunits of 70 kDa (Ku70) and 80 kDa (Ku80 or Ku86) which bind to DNA DSBs ends [19, 20]. This Ku–DNA complex then serves as a platform for the recruitment of additional core NHEJ components. Among the first to be recruited is the DNA-PKcs, a 460-kDa serine/threonine kinase that plays a pivotal role in coordinating and advancing the NHEJ process [19, 20]. On the other hand, proteins taking part in HR include the DSB proteins RAD50, RAD51, and the RAD51 paralogues X-ray repair cross-complementing protein 2 (XRCC2) and XRCC3, in humans [21, 22]. 

A few studies demonstrate that genes involved in DSBR, such as Ku70, RAD50, RAD51, XRCC2 and XRCC3, exhibit differential expression in pterygium when compared to healthy subjects [23–25]. Specifically, RAD50, that promotes the rapid repair of DSBs via HR, is over-expressed in pterygium conjunctiva, suggesting that recombinational DNA repair mechanisms might be highly activated in this disease [24]. In line with this hypothesis, RAD51, XRCC2 and XRCC3, are elevated in peripheral blood lymphocytes of patients with the history of pterygium recurrence [24]. In addition, PLK1 (polo-like kinase 1), which regulates the activity of RAD51 gene expression, is also highly expressed in pterygia [26]. Moreover, Tsai and colleagues demonstrated that the T-991 C polymorphisms (rs5751129) of Ku70 promoter, the protein playing an initial function in DSB repair by NHEJ, is associated with genetic predisposition to pterygium [25]. 

The aim of this study was to investigate alterations of the main complex involved in DSB repair via the NHEJ in pterygium, by evaluating the expression of DNA-PKcs, Ku70 and Ku80 in both pterygium and healthy conjunctival tissues.

## Materials and methods

### Patient population

Six patients with recurrent pterygium (4 females and 2 males; age 35–71 years) and six age- and sex- matched controls (4 females and 2 males; age 40–73 years) were included in this prospective interventional study, conducted at Sapienza University of Rome, Italy, between February 2024 and April 2025. Among the pterygium patients, two were Caucasian and four were hispanic; among the controls, three were caucasian three were hispanic.

All pterygium patients included in the study underwent surgical excision for recurrent disease. All recurrent lesions were located nasally. The control group consisted of patients undergoing cataract surgery or scleral buckling surgery for rhegmatogenous retinal detachment, with no history of pterygium, prior conjunctival surgery, ocular surface disorders, or systemic conditions affecting the ocular surface. Conjunctival tissue samples were collected intraoperatively during pterygium excision in patients, and as conjunctival biopsies obtained at the time of cataract extraction or retinal surgery in controls. In both groups, all topical ocular treatments were discontinued at least 15 days prior to surgery, and none of the participants were receiving treatment for any other ocular condition at the time of tissue collection.

### Histology and immunofluorescence

Following conjunctival biopsy from control donors or surgical excision of pterygium tissue, samples were fixed in 10% neutral buffered formalin for up to 18 h. Tissues were then embedded in paraffin and sectioned at 4 μm thickness using a microtome. Sections were processed for light microscopy with hematoxylin-eosin staining [27] and for immunofluorescence analysis targeting DNA-PKcs.

For immunostaining, sections were incubated overnight at 4 °C with a mouse monoclonal primary antibody against DNA-PKcs (Ab-32566, 1:300 dilution; Abcam). After rinsing in PBS, slides were incubated for 30 min at room temperature with a fluorescently labelled secondary antibody (1:250; Thermo Fisher Scientific) [28]. Nuclei were counterstained with DAPI [29]. Samples were mounted using ProLong™ Glass Antifade Mountant (Thermo Fisher Scientific) and analyzed using a Zeiss Airyscan confocal fluorescence microscope at 40× magnification.

### Sample preparation for SDS-PAGE and western blot analysis

Conjunctival samples were minced in 10 volumes of RIPA buffer (50 mM TRIS HCl pH 7.5, 150 mM NaCl, 1% Triton X-100, 0.5% sodium deoxycholate, 0.1% SDS, 0.1 mM dithiothreitol, 1 × protease inhibitor, 1 × phosphatase inhibitor) and homogenized with a tight Potter-Elvehjem until a clear solution was obtained. Unsoluble material was precipitated at 15.000 × *g* for 15 min at 4 °C. Supernatants (1–3 µl) were assayed for protein concentration through Micro BCA™ Protein Assay Kit (Thermo Fisher Scientific). Appropriate amounts of protein (10–30 µg) extracts were boiled for 5 min in SDS-PAGE Laemmli buffer (50 mm Tris-HCl, pH 6.8, 2% SDS, 10% glycerol, 0.1% bromphenol blue, 50 mm DTT) and separated by SDS-PAGE (5% polyacrylamide for DNA-PKcs, 10% for Ku70, Ku86, Catalase, total AKT and β-actin). Proteins were electrotransferred to nitrocellulose membrane (Hybond™ C-extra, GE Healthcare) at 30 V overnight at 4 °C for DNA-PKcs detection and at 100 V for 1 h at 4 °C for the other proteins analyzed.

Membranes were blocked for 1 h RT with 10% (w/v) milk in TBS-T solution (Blocking buffer, 0.1% Tween 20 in 1.3 m NaCl, 200 mm KCl, 250 mm Tris, pH 7.5) and incubated with primary antibodies using the following dilutions: anti-β-Actin 1:1000 (Sigma A3853); anti-DNA-PKcs Ab-4 mixture 1:400 (Neo Markers MS-423-P); anti-Ku70 1:400 (Santa Cruz Biotechnology sc1486); anti-Ku86 1:500 (Santa Cruz Biotechnology sc1484); anti-Catalase 1:1000 (Cell Signaling Technology, UK); anti-pan AKT (Cell Signaling Technology, UK) in TBS-T containing 2 or 5% milk for 2 h RT or overnight at 4 °C with gentle shaking.

After extensive washing in TBS-T, membranes were probed for 1 h RT with HRP-conjugated antibodies (anti-rabbit IgG 1:100,000 (711-035-152) and anti-mouse IgG 1:100,000 (715-035-151) Jackson ImmunoResearch; anti-goat IgG 1:500,000 (sc-2768) Santa Cruz Biotechnology) diluted in TBS-T containing 2% milk and washed thoroughly with TBS-T. Immunoreactive bands were detected using an enhanced chemiluminescence (ECL) detection system (EuroClone).

Images were acquired using an Amersham ImmageQuant 800 (GE Healthcare), and densitometric analysis was performed using ImageQuant software (GE Healthcare) and Fiji Image software (NIH).

### Real-time PCR

Total RNA from conjunctival samples (Healthy *n* = 3; Pterygium *n* = 4) was extracted using Trizol reagent (Invitrogen, Milan, Italy) separated on a 2% agarose gel to confirm integrity of purification and quantified by densitometric analysis using the Fluor-S system, Bio-Rad (Milan, Italy). Total RNA (1 µg) was reverse transcribed using the first-strand synthesis system for RT-PCR (Superscript, Invitrogen) in 20 µl final volume for 1 h at 42 °C following the manufacturer’s protocol.

PCR amplification was achieved using primers specific for human DNA-PKcs: forward 5′-CTTTCTTTCCACCCTTTGTCTC-3′ and reverse 5′-AGCAGAGCCTCCTCTAGCAG-3′, spanning exons 62–63 of the human PRKDC gene. Primers for 18 S rRNA (forward 5′-GTAACCCGTTGAACCCCATT-3′; reverse 5′-CCATCCAATCGGTAGTAGCG-3′) were used for relative quantification.

Relative quantitative Real-Time PCR was performed in a Real-Time Thermocycler (MX 3000, Stratagene, Milano, Italy) using the Brilliant SYBR Green QPCR Master Mix according to manufacturer’s instructions. The dye ROX (Stratagene) was included in each reaction to normalize non-PCR-related fluctuations in fluorescence signal. All PCR reactions were coupled to melting-curve analysis to confirm the amplification specificity. Specific amplification was confirmed by ethidium bromide staining of the PCR products on agarose gel. Non template controls were included for each primer pair to check for any significant levels of contaminants.

### Statistical analysis

Statistical analyses were performed using GraphPad Prism (GraphPad Software, San Diego, CA, USA). Continuous variables are presented as mean ± standard deviation (SD). Comparisons between healthy conjunctiva and recurrent pterygium samples were performed using unpaired two-tailed statistical tests. For Western blot densitometric analyses, differences between healthy conjunctiva and recurrent pterygium were evaluated using Welch’s t-test. For RT-PCR experiments, gene expression levels were calculated after normalization to the reference gene (18 S rRNA), and differences between groups were assessed using an unpaired Student’s t-test. P value < 0.05 was considered statistically significant.

## Results

**Fig. 1:**
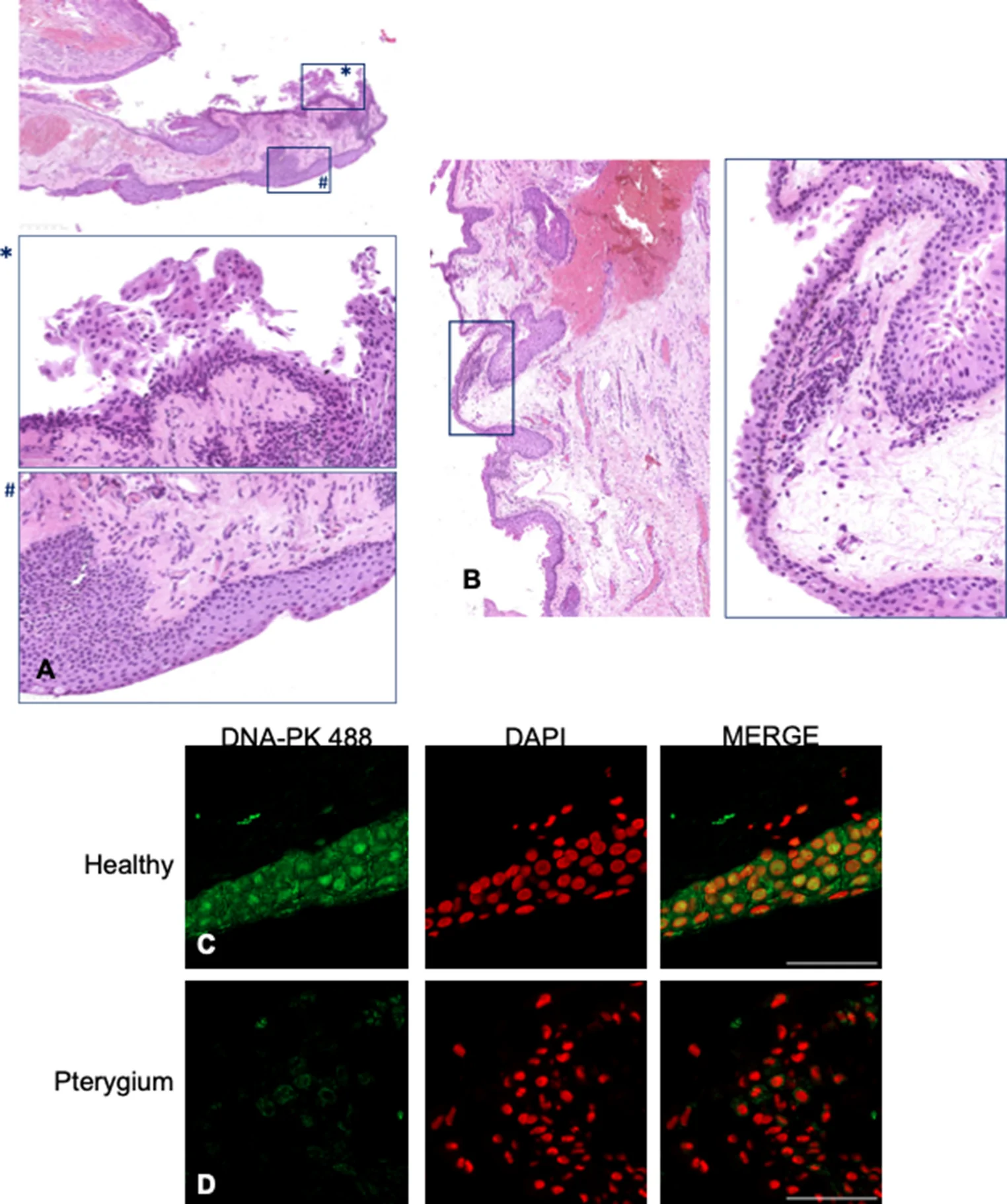
DNA-PKcs is markedly reduced in pterygium conjunctiva

We first confirmed the diagnosis of pterygium in all recruited patients by histological analysis. Conjunctival specimens revealed subepithelial fibrovascular proliferation with vermiform or elastotic changes, focal disruption of Bowman’s layer by fibrovascular ingrowth, and an overlying epithelium of normal morphology (Fig. 1A and B). In some cases, the conjunctival epithelium exhibited acanthosis, hyperkeratosis, or dysplastic alterations, accompanied by goblet cell loss and areas of goblet cell hyperplasia (Fig. 1A).

To assess the involvement of the DNA repair protein DNA-PKcs in pterygium, we examined its expression and distribution in conjunctival tissues from patients and healthy controls. Immunofluorescence analysis was performed on all specimens, yielding consistent results; representative images are shown in Fig. 1C and D. In healthy conjunctiva, DNA-PKcs displayed strong immunoreactivity throughout the epithelial layer, with sparse staining in the subepithelial stroma (Fig. 1C). In contrast, pterygium conjunctiva exhibited a marked reduction of DNA-PKcs labeling across all epithelial layers (Fig. 1D).

Representative images of pterygium conjunctiva stained with hematoxylin and eosin.

(A) Upper panel: low magnification image showing fibrovascular proliferation composed of collagen mixed with blood vessels. Epithelial changes show squamous metaplasia with goblet cell loss and acanthosis (* and #). (B) Images of pterygium conjunctiva stained with hematoxylin and eosin showing sub-epithelial chronic inflammatory infiltrate (low and high magnification). (C and D) Representative double immunofluorescence images of conjunctival epithelium labelled with an anti-DNA-PKcs antibody (green channel) and DNA dye (red channel). Healthy conjunctiva shows strong DNA-PKcs expression (C), whereas pterygium tissue displays markedly reduced labeling (D). Scale Bar 50 μm.

**Fig. 2:**
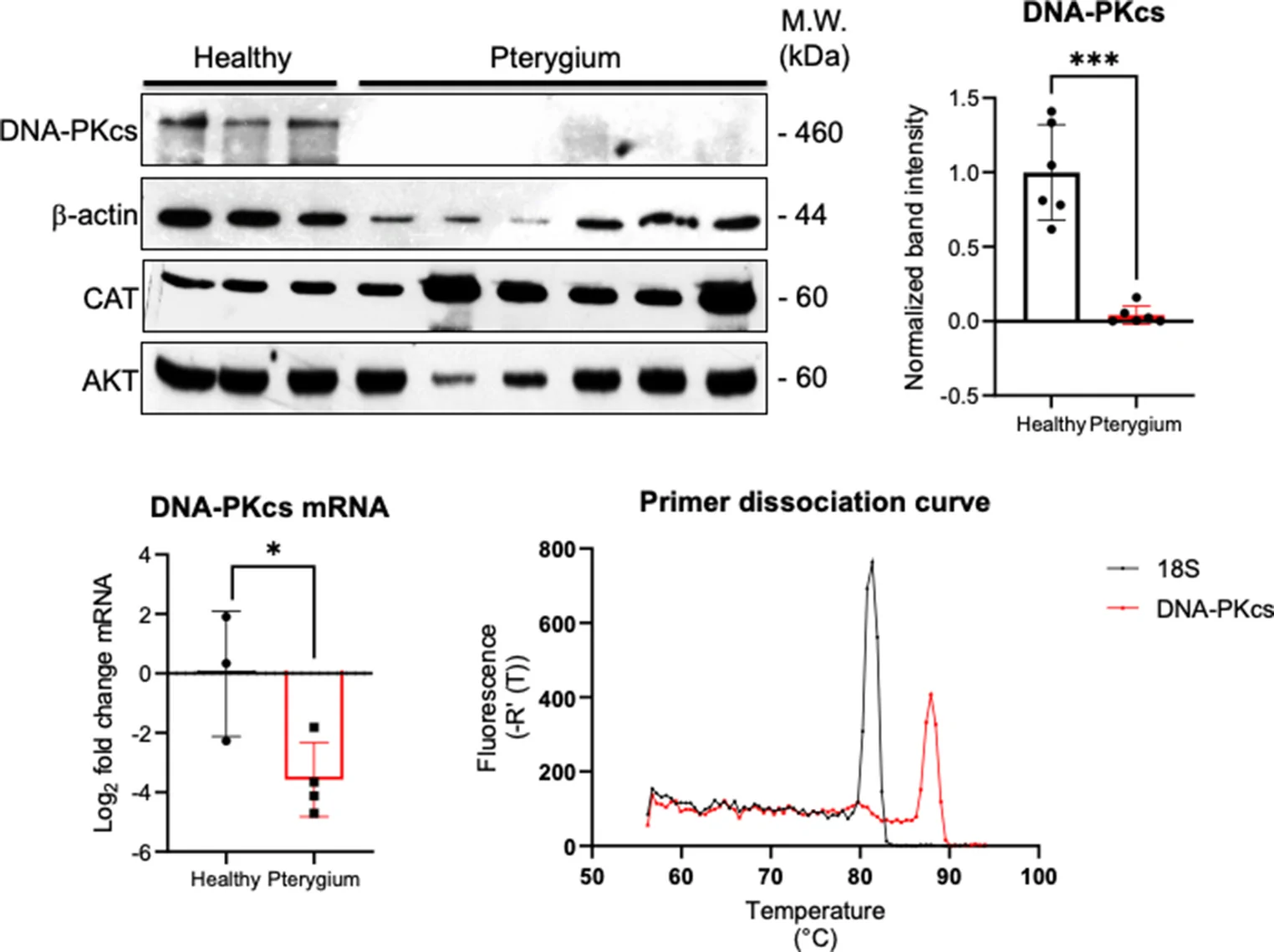
DNA-PKcs protein and mRNA levels are markedly decreased in pterygium conjunctiva

To quantify DNA-PKcs protein levels in pterygium conjunctiva, we performed Western blot analysis. DNA-PKcs was strongly expressed in healthy conjunctiva but was barely detectable in pterygium samples (Fig. 2, upper left panel). Densitometric quantification of band intensity, normalized to β-actin, confirmed a highly significant reduction of DNA-PKcs in pterygium compared with healthy controls (Fig. 2, upper right panel; ***p < 0.001).

To determine whether the reduction of DNA-PKcs protein in pterygium reflected transcript downregulation rather than protein instability, we performed real-time PCR analysis. DNA-PKcs mRNA levels were significantly decreased in pterygium compared with healthy conjunctiva, with an approximately 13-fold reduction (Pterygium: − 3.572 ± 1.25; Healthy: − 0.011 ± 2.11, log₂ values) (Fig. 2, lower left panel). The dissociation curve confirmed primer specificity (Fig. 2, lower right panel). These results indicate that the loss of DNA-PKcs protein is associated with impaired mRNA expression in pterygium.

Upper panel: Representative Western Blot analysis showing the expression of DNA-PKcs protein in different healthy subject conjunctiva and its absence in patients affected by pterygium. b-actin was used as a loading control along with AKT and Catalase. Histogram data represent the normalized DNA-PKcs band intensity in healthy (*n* = 6) and pterygium conjunctiva (*n* = 6). Statistics by Welch’s test, ***p < 0.001. Bars in the plots represent means ± SD.

Lower panel: Real-time PCR of RNA isolated from healthy and pterygium affected conjunctiva. Histogram represents DNA-PKcs mRNA values plotted as log₂ fold change to control. 18 S rRNA expression was used for each sample normalization. Significant reduction is observed for DNA-PKcs transcript. Statistics by Student’s t-test (unpaired), * *p* < 0.05. Bars represent means ± SD. The dissociation curve plot shows target specificity for both primer sequences.

**Fig. 3:**
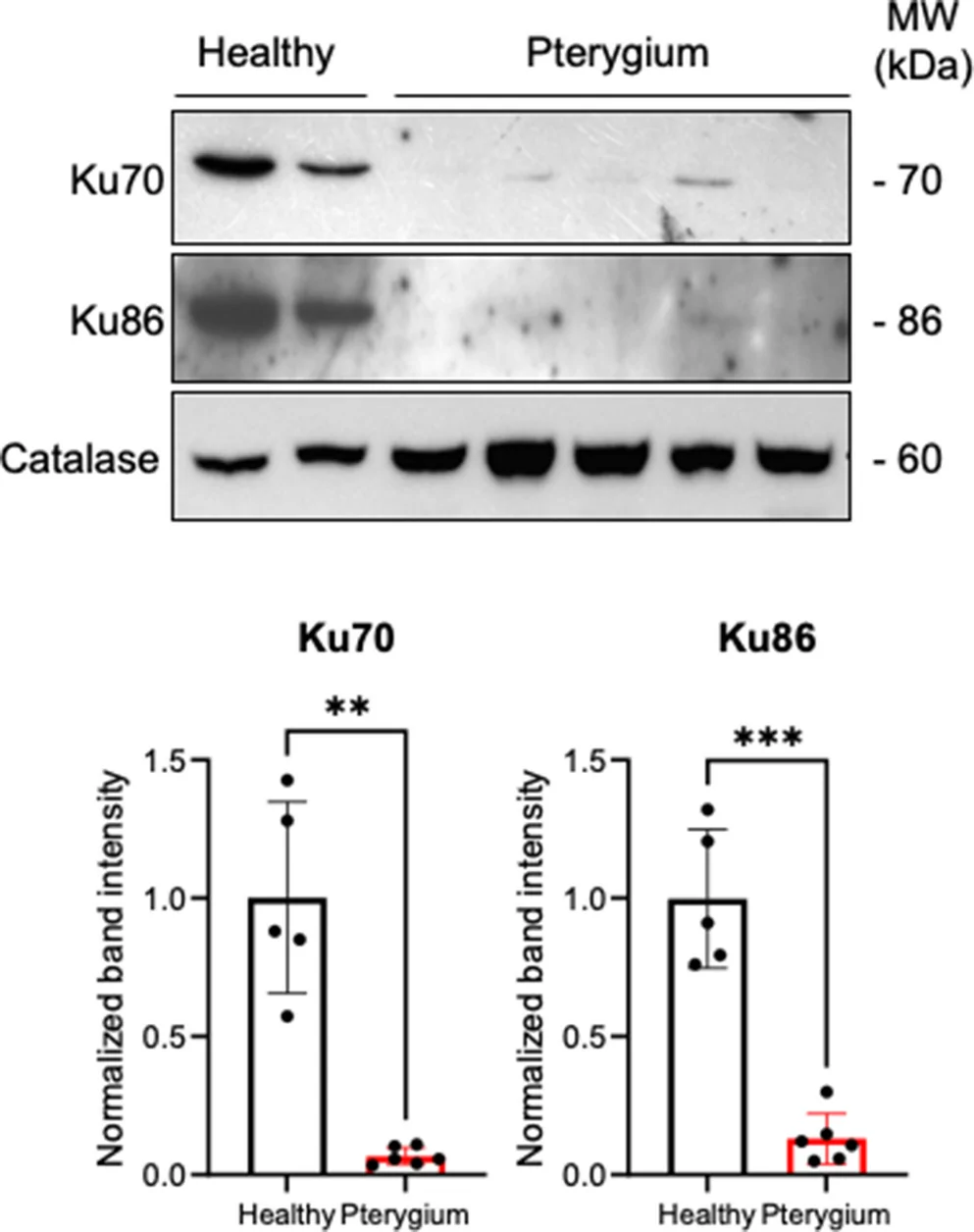
DNA-PK complex proteins Ku70 and Ku 86 are markedly reduced in pterygium conjunctiva

We then analyzed whether pterygium conjunctiva also showed a modulation of the DNA-PKcs complex regulatory subunits Ku70 and Ku86. Densitometric analysis of immunoreactive bands showed that both proteins were strongly reduced in patients as compared with healthy subjects (Fig. 3). Specifically, Ku70 expression was decreased by 93.5% (Healthy: 1 ± 0.34; Pterygium: 0.065 ± 0.029), while Ku86 expression was reduced by 92.0% (Healthy: 0.998 ± 0.251; Pterygium: 0.128 ± 0.091).

These results indicate that the integrity of the DNA-PK complex is profoundly compromised in pterygium, suggesting a mechanism of dysregulation that warrants further investigation.

Representative Western Blot analysis of DNA-PK complex proteins Ku70 and Ku86 showing their expression levels in different healthy subject (*n* = 6) conjunctiva and its reduction in conjunctiva affected by pterygium (*n* = 6). Catalase was used as a loading control. Histogram data represent the normalized band intensity in healthy and pterygium conjunctiva. Statistics by Welch’s test, **p < 0.01; ***p < 0.001. Bars in the plots represent means ± SD.

## Discussion

This is the first study showing a marked reduction of proteins involved in DNA DSB repair via the NHEJ in the conjunctiva of patients with recurrent pterygium. Here we highlight, by immunofluorescence and molecular analysis, the lack of DNA-PKcs as well as its regulatory subunits Ku70 and Ku86 in the conjunctiva of pterygium patients compared to control conjunctiva.

Although the pathogenesis of pterygium remains unclear, several risk factors have been proposed, including UV exposure [2, 4, 5, 9], age [2], race [2] and family history [2, 6]. Among these, UV radiation is the most consistently associated with pterygium development, but the molecular mechanisms underlying UV-induced disease remain elusive [2]. 

One possible explanation is that chronic UV exposure generates reactive oxygen species (ROS), leading to oxidative stress and DNA damage. Impaired DSB repair is a well-recognized feature of ROS-related disorders, including neurodegenerative diseases and cancer [13, 16, 19, 24]. In this context, pterygium may be regarded as a proliferative disease driven by ROS generated through long-term sunlight exposure and resulting chronic oxidative stress. Specifically, ROS produced following UVA absorption by cellular chromophores can induce oxidative DNA damage, ultimately leading to both single- and double-strand breaks [7, 13, 16, 17, 20, 30]. 

The association between pterygium and lack of Ku70, Ku86 and DNA-PKcs strongly suggests that ROS-dependent impairment in DSB repair is a critical event in the development of pterygium. Accordingly, our previous study demonstrated that oxidative stress mediated by aggregated Aβ impairs DNA-PK activity through ROS production, leading to DNA-PKcs degradation [10, 11, 31]. Consistent with our findings, a Ku70 gene polymorphism has been reported in patients with pterygium [25]. Additional evidence indicates that a polymorphism in HOGG1, a key gene in the base excision repair pathway, is associated with pterygium development, further supporting the hypothesis of an impairment of DNA repair system in pterygium patients [30, 32]. Moreover, dysfunction of p53, a central regulator of cell fate following DNA damage, has been described in pterygium [33] providing additional evidence that this condition represents a UV-related proliferative disorder driven by defective DNA repair mechanisms.

DNA-PKcs is known to exert multiple functions in mammalian cells [31]. In addition to its canonical role in the NHEJ pathway, it plays emerging roles in cell cycle regulation and mitosis, where its depletion or pharmacological inhibition delays the metaphase–anaphase transition. Accordingly, the loss of DNA-PKcs in pterygium cells may contribute to cell cycle disturbances and defective mitotic progression. Moreover, mutations in the DNA-PKcs gene can promote genomic instability, potentially cooperating in the uncontrolled cell proliferation characteristic of pterygium.

We hypothesize that DNA repair mechanisms are impaired in pterygium and may be disrupted at multiple levels, contributing to the well-documented heterogeneity in clinical presentation, disease progression, and surgical outcome. These alterations could form the basis for future clinical studies aimed at identifying novel biomarkers. Given that our data show that lower levels of DNA-PK are detected in recurrent pterygium compared to normal conjunctiva, and that DNA-PKcs has been shown to modulate transcriptional networks that induce cell migration, invasion and metastasis [7, 10], we suggest that evaluation of Ku70, Ku 86 and DNA-PKcs expression in pterygium conjunctival tissue may provide valuable biomarkers for predicting pterygium susceptibility and for guiding targeted therapeutic approaches.

Several limitations of the present study should be acknowledged. The relatively small sample size may limit statistical power. In addition, analyses were performed on whole-tissue extracts, which limits the exact discrimination between cell-intrinsic regulation of DNA-PK complex components and differences driven by cellular proportions. Although immunofluorescence supported reduced epithelial DNA-PKcs expression in recurrent lesions, this approach does not provide cell-type quantification. Furthermore, only recurrent pterygium specimens were analyzed. While recurrence represents a model of persistent pathogenic activity in the setting of chronic UV-induced DNA damage, primary and recurrent lesions may exhibit distinct molecular profiles. Comparative studies including both primary and recurrent lesions will be necessary to determine whether NHEJ pathway impairment represents an early pathogenic event or a feature associated with recurrence.

In conclusion, this study provides the first evidence that DNA-PK complex is associated with the development of pterygium, suggesting a possible relationship between pterygium pathogenesis and DNA DSBs. The exact nature of this association, however, requires further investigation. In view of the complexity in the aetiology of pterygium, NHEJ proteins might be incorporated into a panel of susceptibility biomarkers to identify individuals at increased risk of developing the disease.

## Supplementary Information

Below is the link to the electronic supplementary material.

Supplementary Material 1 (PDF 736 KB)

## Data Availability

No datasets were generated or analysed during the current study.
