# Supplementary material for: Loss of DNA-PK complex in recurrent pterygium suggests a role for defective double-strand break repair
Source: Graefes Arch Clin Exp Ophthalmol. 2026 May 26;264(11):3663–71. doi: 10.1007/s00417-026-07283-4 (PMC13736378; doi:10.1007/s00417-026-07283-4)

Original Western blots for Figure 2

Crop used in figure

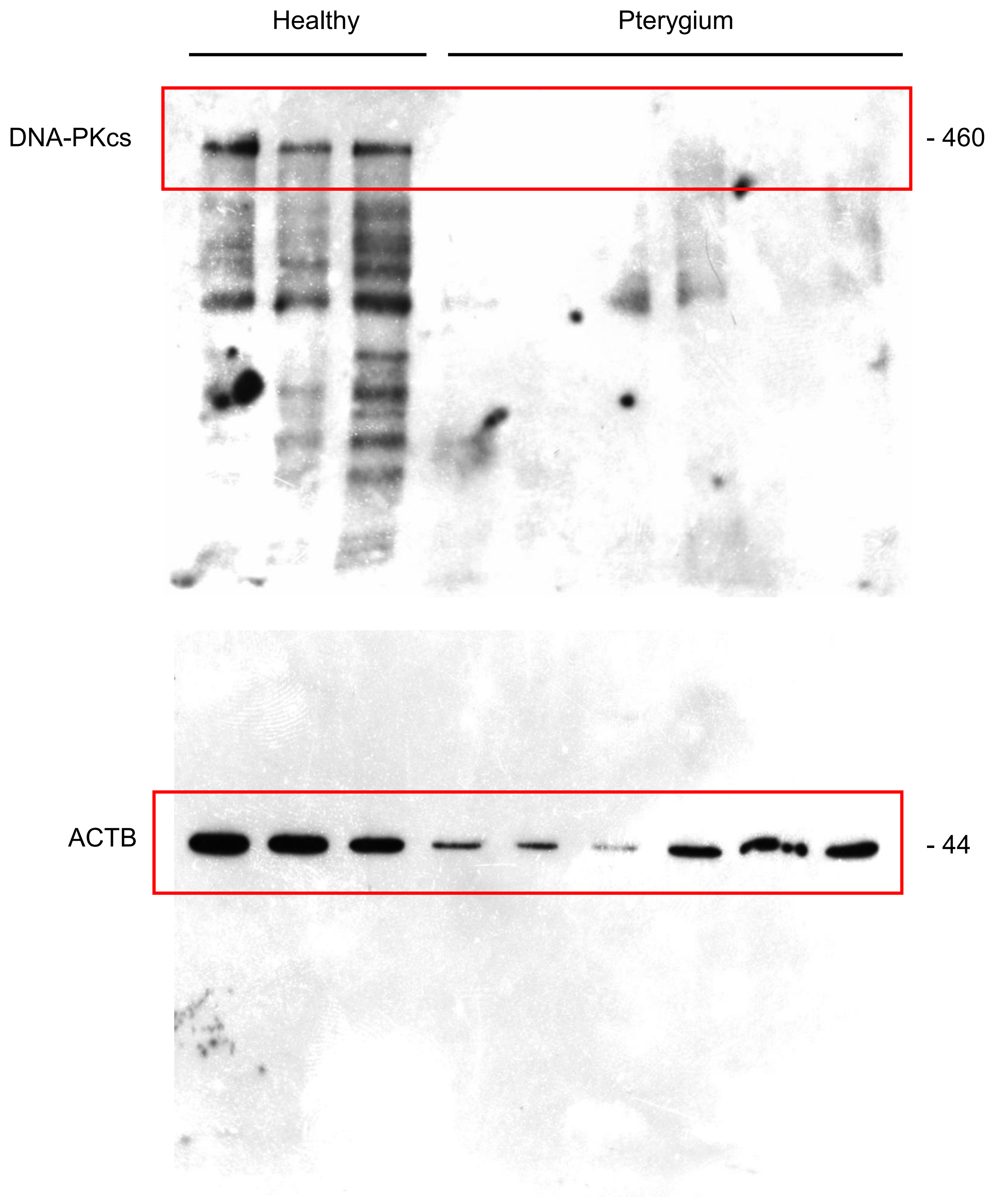

Original Western blots for Figure 2

Crop used in figure

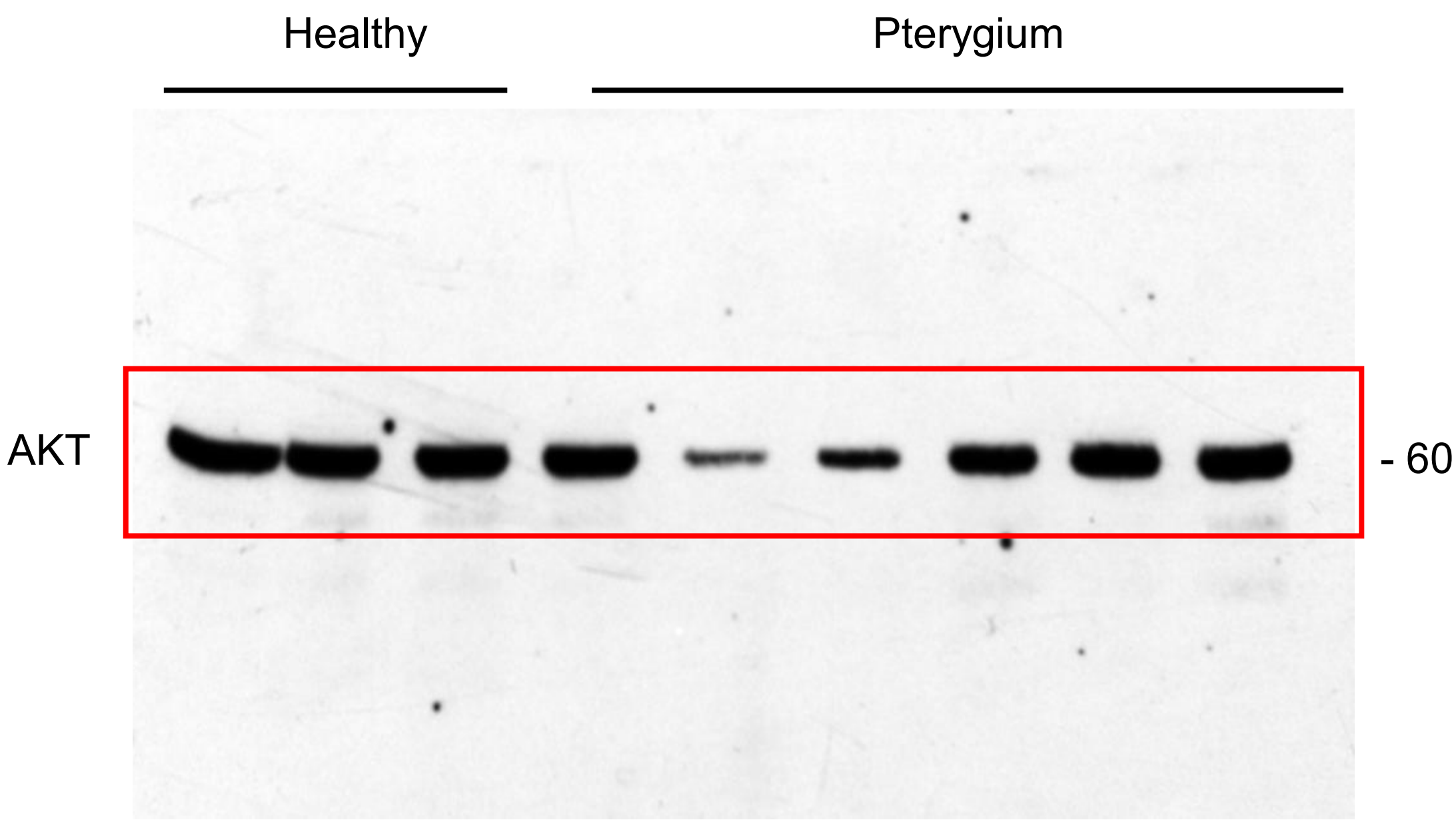

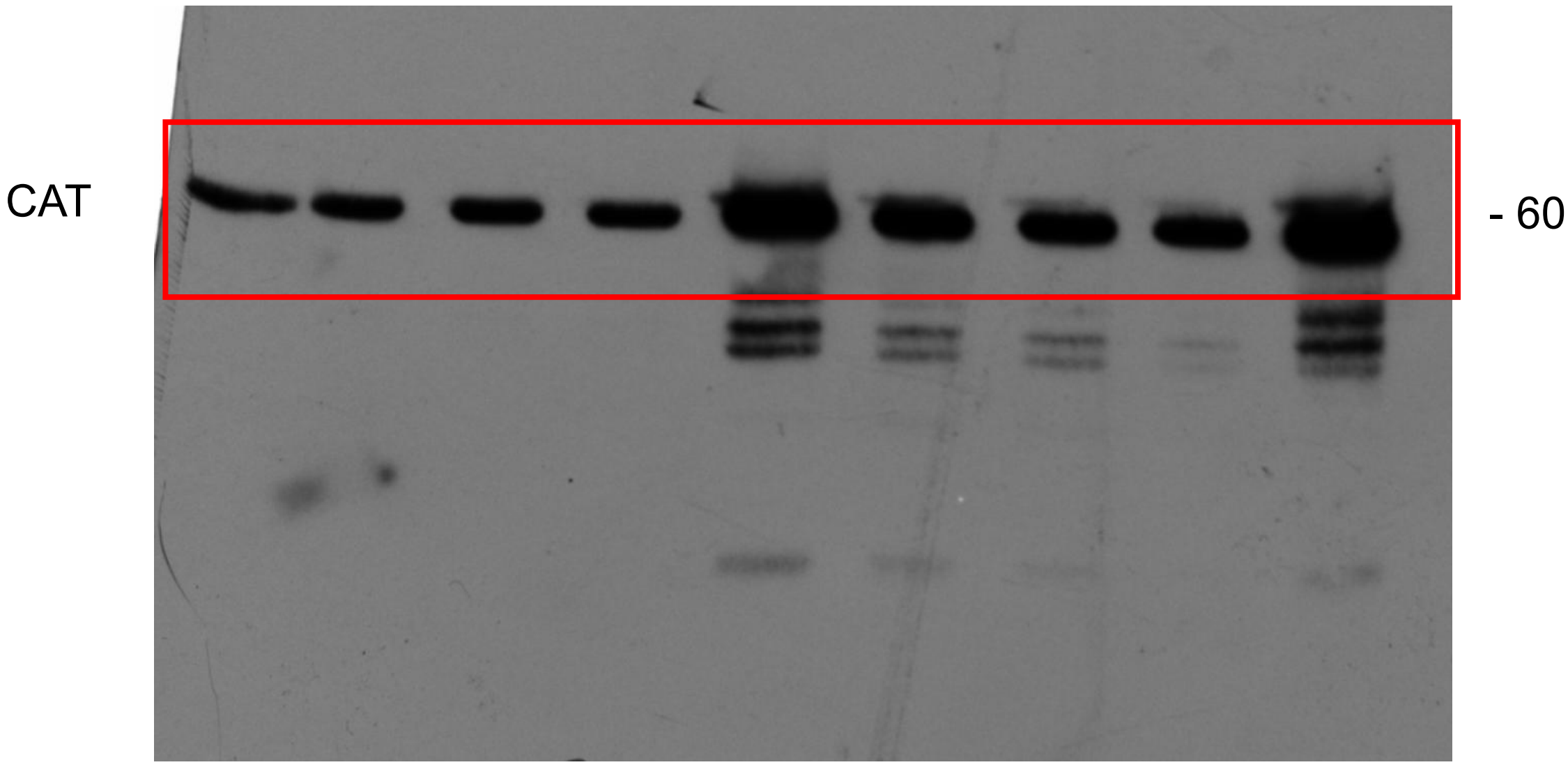

Original Western blots for Figure 3

Crop used in figure

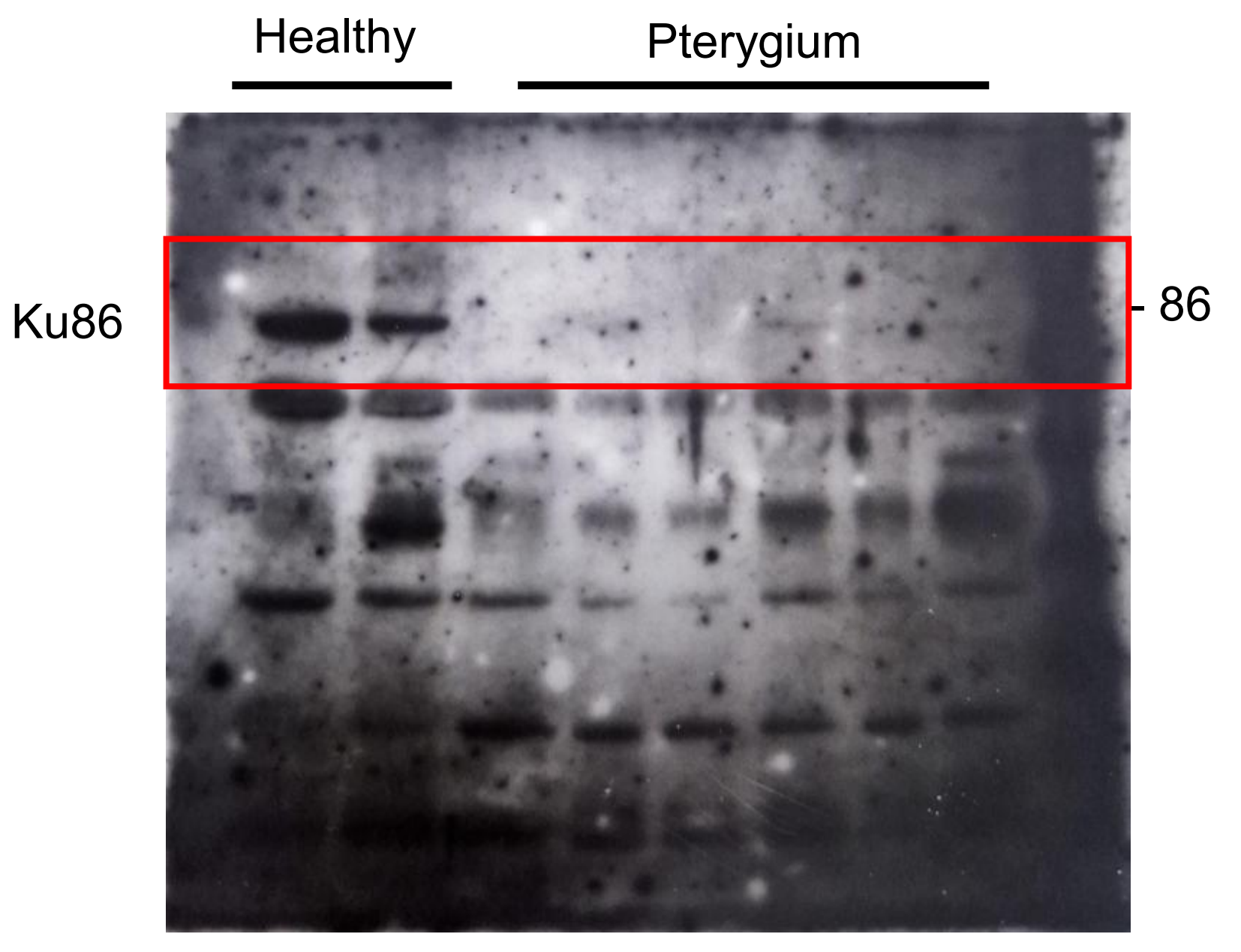

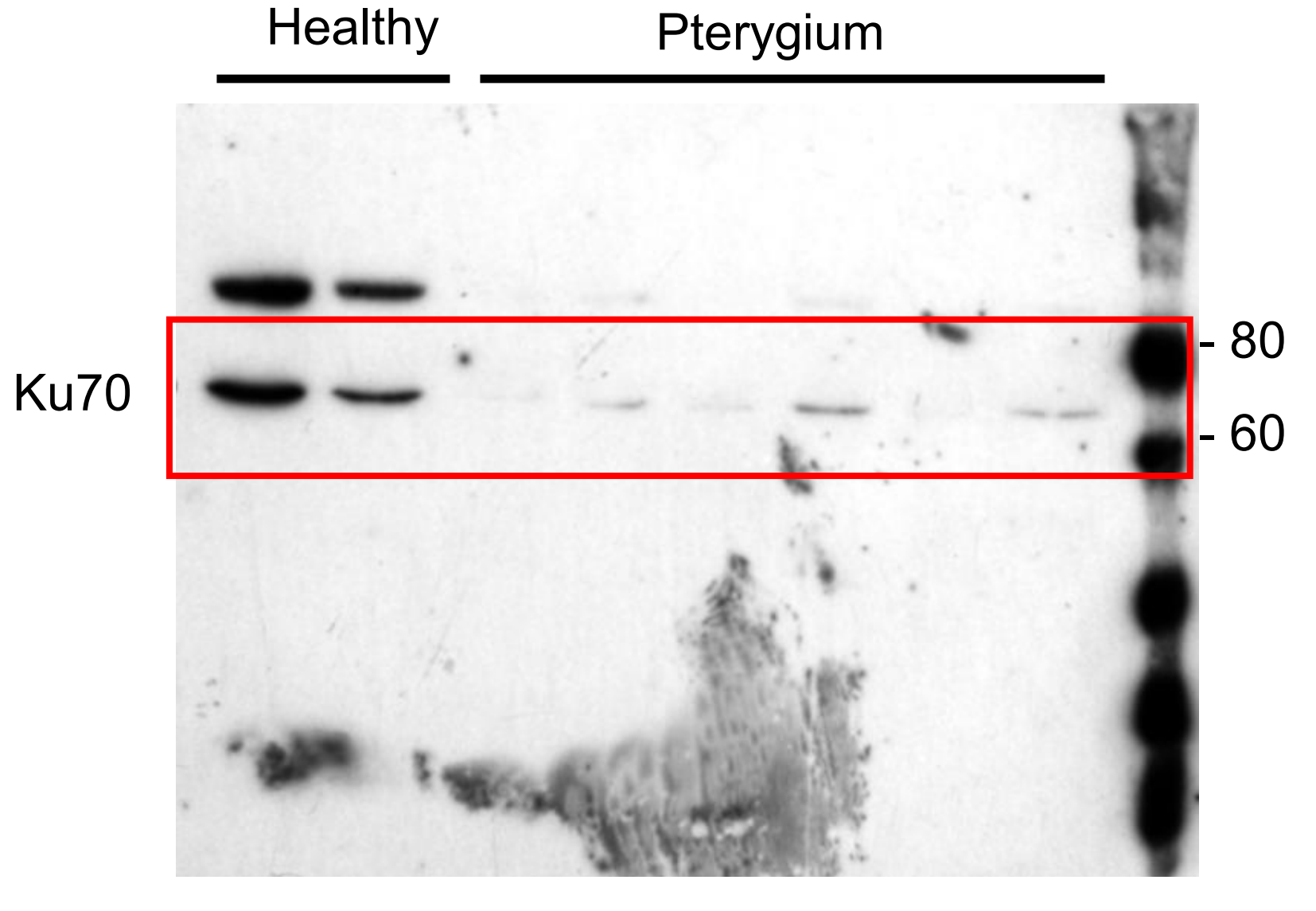

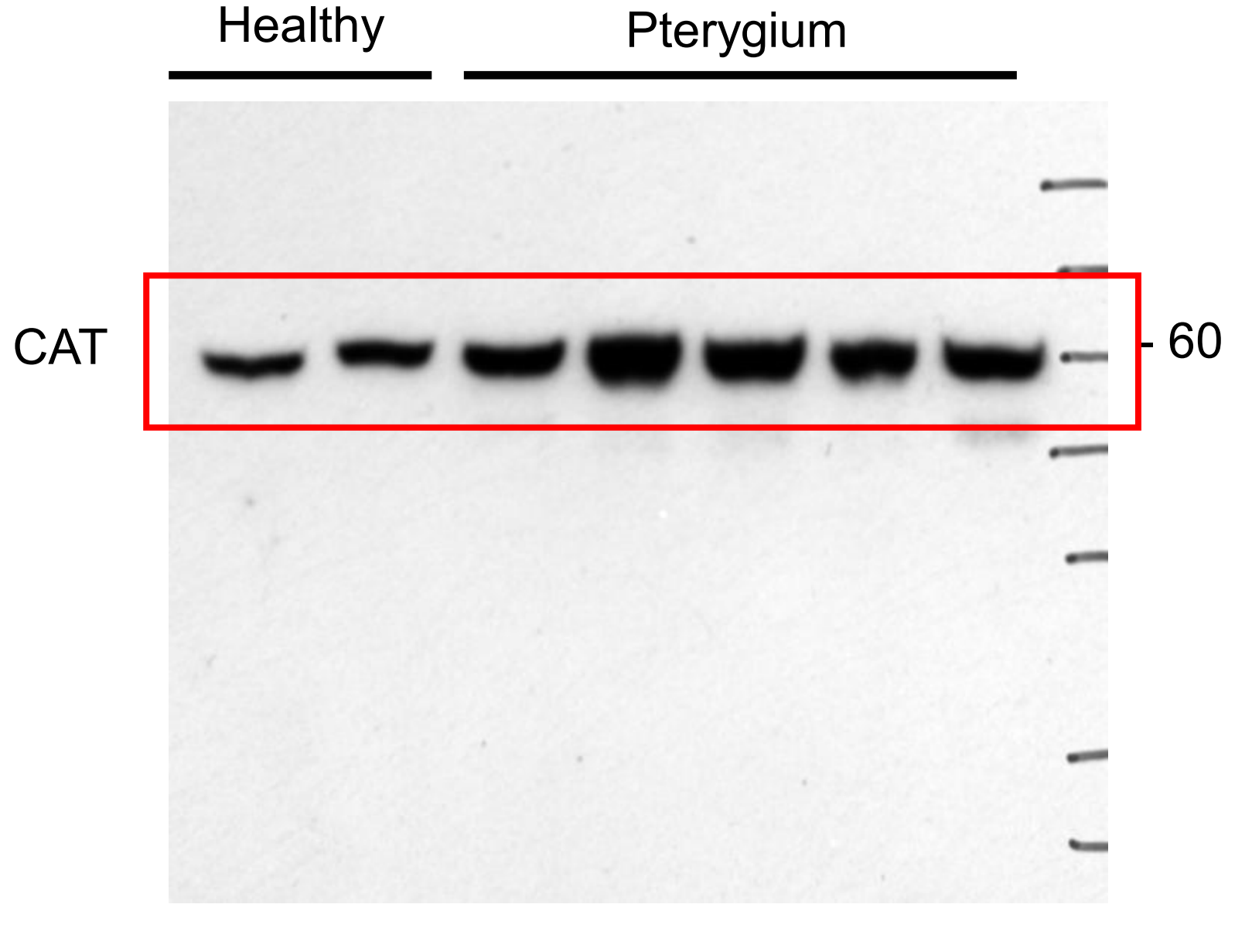

Supplement: Supplementary file 1 [file 417_2026_7283_MOESM1_ESM.pdf]
